# Optimisation of Micronutrient Supplementation During Pregnancy: Interactions Between Bioactive Compounds, Their Impact on Health, and Regulatory Considerations

**DOI:** 10.3390/biom16040540

**Published:** 2026-04-05

**Authors:** Rebeka Anna Makó, Péter Sipos

**Affiliations:** 1 Doctoral School of Food Sciences, Faculty of Agricultural and Food Sciences and Environmental Management, University of Debrecen, Böszörményi Street 138, 4032 Debrecen, Hungary; 2 Institute of Nutrition, Faculty of Agricultural and Food Sciences and Environmental Management, University of Debrecen, Böszörményi Street 138, 4032 Debrecen, Hungary

**Keywords:** pregnancy, food supplements, consistency of preparations, micronutrients, bioavailability

## Abstract

The intake of vitamins and minerals has a significant impact on the health of both the expectant mother and the newborn. During gestation, the demand for micronutrients increases; therefore, modifying dietary habits and selecting foods that ensure adequate (or sufficient) intake can be challenging. Although food supplements contain substances with beneficial physiological effects and their use can improve micronutrient intake, these products cannot replace proper nutrition. Due to modern nutritional habits, intake of key micronutrients is often inadequate, and their deficiencies are known to correlate with significant clinical outcomes during pregnancy. To reduce these deficiencies, several single- and multi-component dietary supplements have been developed. This review aims to present the health effects of the bioactive compounds found in these products and to discuss interactions (i.e., synergistic, additive, and antagonistic effects) between the micronutrients that may alter their bioefficiency. In addition, with a focus on future directions, this review draws attention to the need for a reassessment of current nutritional guidelines and recommendations, the development of new approaches, and emphasises the importance of establishing harmonised amounts of vitamins and minerals used in dietary supplements for pregnant women.

## 1. Introduction

In certain special conditions, such as pregnancy, the demand for several vitamins and minerals increases significantly [1,2]. Furthermore, considering the fact that the risk of inadequate intake of certain micronutrients (such as iron, iodine, folic acid, and vitamin D) is high across Europe, targeted supplementation may become essential during this period [3,4,5]. Optimising micronutrient intake is the most effective way to prevent micronutrient deficiencies. In order to ensure that nutritional goals are achieved, it is essential to modify eating habits, diversify the diet, and use food supplements effectively. If a pregnant woman cannot consistently improve her diet compared with the pre-pregnancy period, she is not capable of achieving a balanced, optimal intake. As a result, she cannot cover the body’s energy and nutrient needs, which may negatively affect both her own health and that of her offspring [6].

Micronutrient deficiencies (such as inadequate levels of vitamin D and calcium) during the gestational period can contribute to adverse pregnancy outcomes and complications, including gestational diabetes mellitus (GDM) and preeclampsia (PE) [7,8]. In terms of foetal complications, these deficiencies significantly increase the risk of intrauterine growth retardation (IUGR) and preterm birth [9]. Furthermore, it is known that the factors listed above may contribute to low birth weight (LBW) and, in several cases, to perinatal loss and intrauterine foetal death [10]. In addition to the fact that maternal deficiency can significantly impair the survival of the foetus and the newborn, it can also have longer-term effects, as it may disrupt the metabolic homeostasis of the offspring [11]. Given the potential health risks presented, from the perspective of prophylaxis, the accurate assessment of nutritional status and the determination and personalised planning of micronutrient supplementation are of paramount importance [12]. For this reason, addressing hidden hunger in pregnant women may involve the use of prenatal products.

However, it is essential to consider the total intake of vitamins and minerals from all sources, including fortified foods, foodstuffs intended for general consumption, and medicines. Furthermore, it is a fact that the storage and breakdown of certain nutrient components in the body show individual differences. Therefore, the possibility of excessive intake should not be overlooked when supplementing micronutrients, as, in many cases, an unjustified large increase in the dose or frequency of administration can exceed the known upper safe level or tolerable upper intake level (UL), hurting the body [13]. Iodine serves as a pertinent example: while adequate intake before and during early gestation can prevent the development of cretinism, excessive iodine intake during pregnancy can lead to foetal hypothyroidism and goitre [14,15].

**Figure 1:**
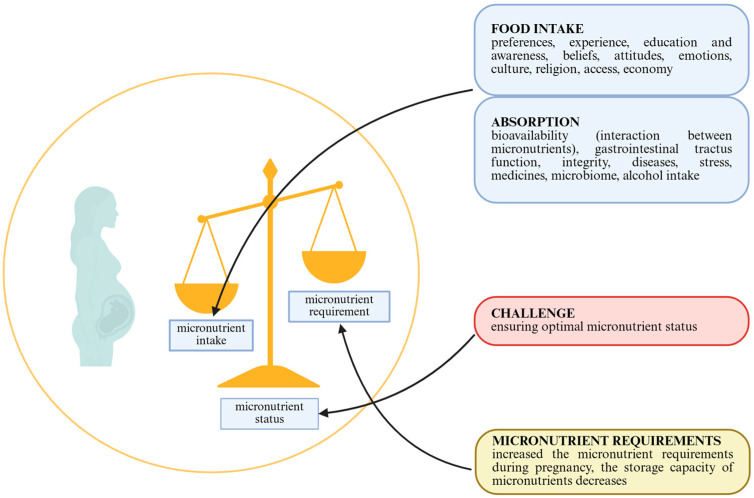
Achieving optimal micronutrient status during pregnancy (Created in BioRender, Mako, R. (2026) https://BioRender.com/0qubh7t, accessed on 23 February 2026).

As maintaining optimal micronutrient status during pregnancy has critical importance, this review aims to offer a comprehensive overview of the appropriate supplementation of key micronutrients required for the normal progression of gestation, which can bring balance between the physiologically determined demands and intake, under the effects of modifying external and internal factors and interactions (Figure 1). It examines the health-related functions of some substances (focusing on iodine, calcium, vitamin D, vitamin B12, folic acid, and iron) present in dietary supplements, elucidates their mechanisms of action, and analyses the interactions among these nutrients and their bioavailability. Furthermore, this review addresses micronutrient requirements during pregnancy in relation to safety considerations, taking into account current recommendations and regulatory guidelines.

## 2. Role of Micronutrients During Pregnancy: Is Supplementation Necessary?

It is important that vitamin and mineral supplementation be tailored to the specific conditions that characterise each of the three main physiological stages of pregnancy. It is known that micronutrients affect implantation during the early stages of pregnancy. Their intake is essential during the embryonic stage of development, a period characterised by histogenesis, organogenesis, and the rapid increase in the size of the embryo due to both cell division and increased cell size [16,17]. Furthermore, adequate micronutrient intake during the foetal period supports both maternal health and foetal development by contributing to processes that are integrated across the maternal, placental, and foetal compartments [18]. In general, the distribution of nutrients between maternal and foetal tissues is regulated by the placenta. The transfer of nutrients at the maternal–foetal interface can occur in several ways, including both passive and active transport [19]. Most water-soluble vitamins presumably enter the foetus via active transport, as the concentration of these substances in the foetal circulation is higher than in the maternal circulation [20]. In the case of minerals, the passage of potassium, magnesium, iron, and calcium across the placenta also occurs through the active transport system [21,22].

The body’s need for calcium increases during pregnancy, especially in the third trimester, as this mineral is essential for the normal mineralisation of the foetal skeleton and for numerous cellular functions, including signal transduction, neurotransmitter release, and cell growth [23]. Maternal adaptive changes, such as decreased renal calcium excretion, increased resorption of calcium from maternal bones, and enhanced intestinal calcium absorption during pregnancy, all serve to meet the calcium requirements of the developing foetus [24]. The desired calcium level also depends to a large extent on the mother’s vitamin D status, as the active form of vitamin D (1,25-dihydroxyvitamin D3) stimulates the absorption of calcium from the small intestine by inducing synthesis of the Ca-binding protein (CaBP) necessary for the absorption of the mineral [25]. In the absence of vitamin D, only 10–15% of dietary calcium and approximately 60% of phosphorus are absorbed [26]. Based on the available data, vitamin D deficiency is a common phenomenon in pregnant women, with a prevalence of up to 90%, which can cause a disruption in the balance of calcium metabolism [27,28]. An inadequate supply of vitamin D and calcium may also be associated with various prenatal pathological processes. Deficiencies in vitamin D and calcium during pregnancy are associated with both skeletal and various non-skeletal disorders. Moreover, such deficiencies increase the risk of preeclampsia, gestational diabetes mellitus, low birth weight, and preterm birth; severe cases of vitamin D and calcium deficiency may lead to bone loss in the mother and the development of craniotabes or hypocalcaemia in the foetus [29]. Similarly, excessive intake of vitamin D and calcium—although rare—can also result in serious health problems such as hypercalcemia (in which disorders of 1,25-(OH)_2_D catabolism should be considered in the differential diagnosis). This condition can lead to vascular and soft tissue calcification and nephrolithiasis [30,31]. Based on this, maintaining adequate vitamin D levels and an optimal calcium-to-phosphorus (Ca/P) ratio has a significant impact on both the maternal and foetal skeletal systems. In the case of maternal skeletal complications, it may also arise that an inadequate supply of vitamin D and calcium may have an adverse effect on haematopoiesis in the bone marrow [32].

Haematopoietic processes, such as erythropoiesis, are also significantly influenced by the availability of three essential nutrients: iron, vitamin B12, and folic acid. Oxygen is transported in red blood cells by haemoglobin, which constitutes the majority of the body’s haem content. A decrease in haemoglobin concentration is referred to as anaemia. The background of anaemia is multifactorial, but the most common cause is iron deficiency, which complicates the course of nearly 50% of pregnancies worldwide [33]. According to the World Health Organization (WHO), anaemia is defined as a haemoglobin concentration below 110 g/L in the first and third trimesters, and below 105 g/L in the second trimester [34]. However, haemoglobin concentration alone cannot accurately reflect the status of the body’s iron stores. For this reason, other specific biomarkers—for example, serum ferritin levels together with C-reactive protein (CRP) and transferrin, which are responsible for iron transport, as well as transferrin saturation (TSAT) and hepcidin—are required to measure iron status [35]. Iron deficiency anaemia can lead to numerous obstetric complications, as it impairs the maternal immune system and reduces resistance to infections. Its presence increases the risk of preterm birth and low birth weight, may induce foetal hypoxia, cause intrauterine growth restriction, and impair the development of the foetal nervous system [36,37]. Therefore, correcting iron deficiency and replenishing iron stores before the third trimester is important, as neurogenesis is most intense during this period [38]. However, it should also be noted that routine iron supplementation in pregnancy should only be recommended for women who are at risk of or have documented iron deficiency, as excessive iron intake or elevated iron status may negatively impact birth outcomes. Potential consequences include increased oxidative stress, heightened blood viscosity, and alterations in the maternal microbiome [37,39]. Unfortunately, the development of iron deficiency during pregnancy can be influenced by numerous factors, including a persistent imbalance between intake and demand, as well as impaired absorption from the gastrointestinal tract. In the case of haem iron introduced into the body through animal-based foods and non-haem iron provided by plant-based sources, differences in the chemical structure of the compounds affect their absorption and bioavailability. Vitamin C supplementation may enhance the intestinal absorption of non-haem iron; however, this effect is highly dependent on an individual’s diet and the dose of ascorbic acid [40,41]. Tannins found in coffee and tea, as well as phytates present in cereals and nuts, significantly inhibit iron absorption [42,43]. However, the phytate content of plant-based foods impairs the absorption of not only iron but also folic acid [44].

Therefore, when estimating nutrient requirements during pregnancy, it is important to consider all factors that influence micronutrient bioavailability, such as folate. In addition to iron, deficiency of this vitamin is also common and most frequently presents as megaloblastic anaemia during pregnancy. This condition is characterised by reduced production and abnormal maturation of erythroid precursor cells. Megaloblastic anaemia is a consequence of impaired DNA synthesis, which arises from deficiencies or alterations in folate metabolism [45]. Folate deficiency has been linked to adverse outcomes in both mothers and foetuses. Based on the highest level of scientific evidence, an adequate folic acid supply is necessary from the moment of conception to prevent neural tube defects (such as spina bifida, myelomeningocele, anencephaly, and encephalocele, which are among the group of craniospinal anomalies), as neural tube closure is completed by the 28th day of pregnancy [46,47]. However, caution should also be exercised regarding high folate intake, as excessive consumption has been associated with an increased risk of gestational diabetes mellitus and impaired placental development, and it may mask vitamin B12 deficiency [48,49].

Like low folate status, suboptimal maternal vitamin B12 levels can impair neural tube development, increasing the risk of spina bifida in offspring, and can also lead to megaloblastic anaemia and/or neuropsychiatric symptoms [50]. In addition, cobalamin (vitamin B12) is essential for homocysteine metabolism, as it acts as a cofactor in the remethylation of homocysteine to methionine by methionine synthase; consequently, vitamin B12 deficiency may result in severe hyperhomocysteinemia [51]. Although vitamin B12 deficiency is less common than folate deficiency, low cobalamin status may occur in pregnant women, even in non-vegetarian populations, particularly in late pregnancy [52]. There are several methods for assessing deficiencies of these vitamins. For example, homocysteine levels and methylmalonic acid (MMA) concentrations in blood and urine are more sensitive indicators of vitamin B12 status than serum vitamin B12 levels [53]. In the case of folic acid, red blood cell (erythrocyte) folate levels more reliably reflect the body’s folate supply than serum folate concentrations [54].

**Figure 2:**
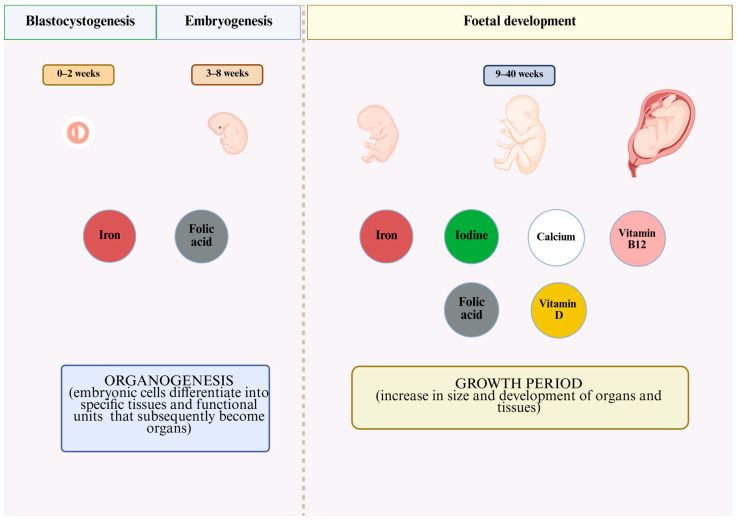
Micronutrients and their function for the development of the offspring during the three main physiological stages of pregnancy (Created in BioRender, Makó, R. A. (2026) https://BioRender.com/ju7o9s1, accessed on 4 April 2026).

However, the requirement for another essential micronutrient, iodine, which is a component of thyroid hormones, also increases significantly during late pregnancy. Iodine deficiency is particularly concerning in pregnant women, taking into account additional needs due to increased maternal thyroid hormone production and iodine uptake by the foetus, placenta, and amniotic fluid. In utero iodine deficiency can result in foetal hypothyroidism and irreversible impairment of cognitive development. Adequate iodine status in pregnant women is indicated by a median urinary iodine concentration between 150 and 499 μg/L [55]. In addition, recent findings suggest that high iodine intake, too, may have negative effects on both maternal thyroid function and pregnancy outcomes [56]. Summarising the above, it is clear that micronutrients play a key role in the stages of pregnancy, and adequate micronutrient status is essential for both maternal and foetal health (Figure 2).

## 3. Micronutrient Requirements for Pregnant Women: European Recommendations

Within the European Union, the European Food Safety Authority (EFSA) has established Dietary Reference Values (DRVs) for vitamins and minerals [57,58]. The term Dietary Reference Values (DRVs) refers to a set of nutrient reference values (including average requirements (ARs), population reference intake levels (PRIs), adequate intake levels (AIs), tolerable upper intake levels (ULs); see Table 1) derived from epidemiological studies, risk assessments, and scientific judgment. However, there is no harmonised methodology for establishing Dietary Reference Values (DRVs). In Europe, most countries have developed their own DRVs, either independently or by adapting existing ones, such as those of the French Agency for Food, Environmental and Occupational Health & Safety (ANSES), the Belgian Superior Health Council (SHC), the Germany, Austria and Switzerland Recommendations (D-A-CH), the Nordic Nutrition Recommendations (NNR), and the Italian Society of Human Nutrition (SINU) [59]. Individual recommendations may differ due to the sources of micronutrient intake and national assessments of needs.

Due to insufficient iron intake in some countries and considering influencing factors such as iron content in food, these countries have decided to recommend a daily supplement. In Denmark, a daily supplement of 40–50 mg of iron is recommended for pregnant women from the 10th week of pregnancy. This recommendation has remained unchanged since 2013 [60]. The iron reference value provided by the 5th edition of the LARN (Livelli di As-sunzione di Riferimento di Nutrienti ed Energia per la popolazione italiana), published by the Italian Society of Human Nutrition (SINU), indicates an AR of 22 mg/day and a PRI of 27 mg/day for pregnant women [61]. In contrast, the European Food Safety Authority (EFSA) indicates a PRI of 16 mg/day of iron intake for pregnant women [62]. Furthermore, some guidelines do not advise routine iron supplementation. In Germany, medical societies do not support prophylactic iron supplementation in pregnancy without a documented iron deficiency [63]. Usually, the amount of elemental iron in iron-containing prenatal supplement products ranges between 9 and 60 mg/serving [64]. However, due to the above factors, increased attention should be paid to iron supplementation during pregnancy.

The need for another common mineral in the body (calcium) also increases during pregnancy. The recommended daily calcium intake is between 900 and 1200 mg. This requirement can be met through calcium-rich foods (such as milk, cheese, nuts, and almonds) as well as supplements, if required. However, it is important to note that most complex prenatal supplements contain only 120–250 mg of calcium [65]. Calcium supplementation of 1500–2000 mg per day may be justified if dietary calcium intake is very low and there is a higher risk of pregnancy complications (e.g., toxaemia of pregnancy) [66,67].

At the same time, adequate vitamin D intake is also necessary for optimal functioning of the body, with a recommended daily intake of 15 μg [57]. To achieve the desired vitamin D level, supplementation should begin in the first trimester of pregnancy. However, loading doses and weekly or monthly administration are not recommended. Based on numerous clinical studies, the maximum daily intake of vitamin D should not exceed 100 μg, even during periods without UV exposure [68,69].

Studies have also shown that folic acid can prevent up to 70% of the previously mentioned neural tube defects when supplementation is used in the periconceptional period [70]. Unfortunately, many women do not consume foods such as legumes, citrus fruits, and leafy green vegetables, which would provide the optimal daily folate intake (about 660 µg) during the periconceptional period and the first trimester of pregnancy. Moreover, studies have shown that the bioavailability of food folate is 50% and that folic acid bioavailability is 85% when taken with food or 100% when taken on an empty stomach with water [71,72]. For this reason, a daily supplementation of 400 μg of folic acid is recommended during the preconception period (at least one month before conception) and throughout the first trimester of pregnancy [53,73]. The maximum daily intake is 1000 µg, and folate intake from food sources (excluding supplements and fortified foods) averages around 300 µg per day, based on European nutritional surveys. Most dietary supplements contain between 100 µg and 400 µg of folic acid per serving, and only about 2% contain 800 µg or more per serving [74]. In some cases, a daily intake of 800 µg may be necessary to achieve optimal red blood cell folate concentrations, defined as values above 906 nmol/L, which are associated with a reduced incidence of neural tube defects (NTDs) [75].

In pregnant women, vitamin B12 deficiency may likewise contribute to the development of such complications. Pregnant women who are at increased risk of vitamin B12 deficiency, particularly those adhering to vegetarian or vegan dietary patterns, should therefore consider appropriate supplementation. The vitamin B12 content of dietary supplements may vary considerably; however, a daily intake of approximately 4.5–5 µg has been proposed [76].

Iodine deficiency is also of particular concern for pregnant women due to their increased iodine requirements during pregnancy. Iodine is found predominantly in animal products, and its sources include fish, seaweed, and dairy products. Therefore, supplementation is necessary for those following specific plant-based diets mentioned earlier. Current guidelines recommend a daily iodine intake of 200–300 μg for pregnant and lactating women to support proper maternal health and foetal development [77].

## 4. Bioavailability of Micronutrients

It is important to recognise that anatomical and physiological parameters change during pregnancy, resulting in altered metabolism and disposition of various active substances from dietary supplements within the body. In this condition, absorption from the gastrointestinal tract is influenced by many factors; for example, gastric pH increases and gastrointestinal motility decreases. The increase in adipose tissue alters the distribution of substances, while increased renal blood flow can accelerate their elimination [78]. Furthermore, the nutritionally important bioactive compounds can be present in various forms and amounts in dietary supplements; therefore, the selection of products and the design of supplementation should focus on relevant considerations, such as the dosage and molecular form of the substances used. This is important because the effects on the maternal organism, placenta, and foetus can vary between individuals, mainly due to differences in the source of supplementation and the heterogeneity of maternal characteristics [79].

### 4.1. Vitamin B9

The term “folate” is generally used to refer to vitamin B9; however, it is important to note that this vitamin is available in both natural and synthetic forms. The biological availability of naturally occurring folate is lower, as discussed earlier, underlining why the concept of dietary folate equivalent (DFE) is important [80]. The synthetic form of folate is called folic acid, which is reduced to dihydrofolate (DHF) by the enzyme dihydrofolate reductase (DHFR) and, through multiple conversion steps, is then transformed in the body into its metabolically active form, L-5-methyltetrahydrofolate (L-5-methyl-THF). If large amounts of folic acid enter the body, the DHFR enzyme can quickly become saturated, leading to the accumulation of unmetabolised folic acid (UMFA) [81]. In contrast, naturally occurring folate in foods exists in the polyglutamate form and is known as tetrahydrofolate (THF). This dietary folate must be further hydrolysed in the intestinal lumen into monoglutamate in order to be rapidly absorbed in the intestine and then enter the portal circulation. The conversion to L-5-methyltetrahydrofolate (L-5-MTHF) depends primarily on the activity of the methylenetetrahydrofolate reductase (MTHFR) enzyme. Therefore, this conversion occurs with reduced efficiency in individuals with an MTHFR gene mutation, which is attributable to the C677T polymorphism in the gene encoding the enzyme [82,83]. The prevalence of this polymorphism depends on geographical location and varies between populations, but averages 30–40% [84]. However, it is not only enzyme function that influences the conversion to biologically active folate, but also the availability of vitamins B2, B6, B12, and zinc. These nutrients are essential for the proper functioning of the folate cycle [85]. Approximately half of the total folate stores of the body (5–10 mg) is stored in the liver, while plasma contains almost exclusively 5-methyltetrahydrofolate (5-MTHF) [86]. Unfortunately, during pregnancy, folate stores can become depleted within three months or even sooner if adequate replenishment is not ensured. This is because pregnancy is characterised by rapid cell division and tissue growth, and the epigenome also changes dynamically during embryonic development to regulate gene expression at different stages of differentiation [87]. An adequate amount of folate is essential for these processes. It has also been established that the bioavailability of folate salts, such as calcium and glucosamine folate, is similar to, or even slightly higher than, that of folic acid. The calcium salt of (6S)-5-methyltetrahydrofolic acid does not require reduction by dihydrofolate reductase and can be directly incorporated into the circulation [88]. Therefore, it is advisable to choose dietary supplements containing these molecular forms [89].

### 4.2. Vitamin B12

Vitamin B12 is a collective term for several structurally similar compounds, all of which contain cobalt. Different ligands can bind covalently to the cobalt atom, resulting in various cobalamin forms. The biologically active coenzyme forms of vitamin B12 are 5′-deoxyadenosylcobalamin and methylcobalamin. Other forms, such as hydroxocobalamin and cyanocobalamin, are inactive, but the body can convert them into the coenzymatically active forms of cobalamin [90,91]. Currently, there is insufficient evidence to suggest that the use of 5′-deoxyadenosylcobalamin and methylcobalamin offers advantages over hydroxocobalamin in terms of bioavailability or biochemical effects. It is important to note that, in cases where a pregnant woman consumes significant amounts of plant-based foods containing cyanogenic glycosides or smokes, leading to the introduction of hydrogen cyanide into the body, it is advisable to choose a supplement containing hydroxocobalamin. This compound can react with cyanide, a toxic compound, converting it into cyanocobalamin and thereby neutralising its harmful effects [92,93]. Cyanocobalamin is also used in certain dietary supplements, likely due to its low cost and heat stability. However, it is not advisable to favour this synthetic form over the naturally occurring forms, as cyanocobalamin must first be broken down into cobalamin and cyanide before it can be converted into its active form in the human body. This process may be inefficient in individuals carrying specific single-nucleotide polymorphisms (SNPs) [94]. For example, it has been established that the common variation in the MMACHC gene, SNP rs12272669, is associated with higher vitamin B12 concentrations in ‘A’ allele carriers compared to ‘G’ allele carriers [95].

### 4.3. Iron

As pregnancy progresses, maternal red blood cell mass increases, and the growth of the placenta and foetus accelerates. Consequently, the physiological requirement for iron rises during the third trimester, reaching approximately 3.0–7.5 mg/day [96]. Unfortunately, many women enter pregnancy without sufficient iron stores to meet the increased demands of this period. In cases of moderate or severe iron deficiency, the entire maternal–placental–foetal unit becomes iron-deficient [35]. For this reason, oral iron supplementation is considered effective for correcting anaemia and replenishing iron stores [97]. A variety of oral iron preparations are available, most of which contain iron in the form of ferrous ions. Accordingly, the most commonly used products contain ferrous salts (Fe^2+^), such as sulphate, fumarate, and gluconate. Oral supplementation with ferrous sulphate or ferrous gluconate may be associated with adverse effects, most notably gastrointestinal irritation, including nausea, epigastric discomfort, constipation, or diarrhoea. However, the severity of these side effects may be reduced and iron absorption improved by administering the supplements on alternate days [98,99]. Nevertheless, it is also well established that ferrous sulphate can increase levels of malondialdehyde (MDA), a terminal product of lipid peroxidation that serves as a biomarker for measuring oxidative stress [100,101]. Despite these factors, iron salts are generally preferred over ferric (Fe^3+^)-containing products, as they exhibit adequate absorption and higher bioavailability [102,103]. Furthermore, for other iron formulations, such as elemental iron (carbonyl iron) and ferrous bisglycinate, clinical data have not demonstrated superiority over ferrous salts. However, ferrous bisglycinate has the advantage of not interacting with dietary phosphates, phytates, or fibre, resulting in faster absorption [104]. Preparations containing ferric hydroxide polymaltose are also commonly used in the treatment of iron deficiency and are characterised by a more favourable side-effect profile compared with ferrous sulphate. However, these formulations are classified as medicinal products and exhibit lower efficacy [105]. Overall, it is important that the effectiveness of iron supplementation becomes evident within 2–4 weeks of treatment initiation, and that oral therapy is continued for an additional 3–6 months, thereby not only correcting the clinical manifestations of iron deficiency but also replenishing iron stores [106]. Treatment of iron deficiency with intravenous iron preparations is warranted only when oral iron supplementation is ineffective or not tolerated by the pregnant woman [107].

### 4.4. Vitamin D

Vitamin D plays an important role in embryonic development, as it is involved in processes such as cell division (proliferation), cell differentiation, and the regulation of angiogenesis. The role of vitamin D in foetal lung development is also well established [20,108]. Increasing evidence suggests that maternal vitamin D deficiency or low vitamin D levels may have long-term effects on the child [109]. In the offspring, attention deficit hyperactivity disorder (ADHD), autism spectrum disorder, and type 1 diabetes mellitus may develop, all of which could potentially be prevented by adequate vitamin D supplementation during pregnancy [27,110,111]. Vitamin D obtained from dietary supplements is biologically inert and must undergo two hydroxylation steps in the body to become physiologically active [112]. In most dietary supplements, vitamin D is present in two main forms—vitamin D_2_ (ergocalciferol) and vitamin D_3_ (cholecalciferol)—which differ only in the chemical structure of their side chains [113]. However, most studies have found that vitamin D_3_ supplementation is preferable to vitamin D_2_, as D_3_ preparations are more stable, and exogenously administered cholecalciferol is better absorbed in the intestine in the presence of bile acids. Vitamin D_3_ increases serum 25-hydroxyvitamin D [25(OH)D] levels more effectively and binds more strongly to the vitamin D-binding protein (DBP), resulting in higher concentrations in adipose tissue, where it is stored [114,115]. Based on these findings, the absorption of cholecalciferol can be considered good. However, scientific data have also led to the recent inclusion of calcidiol monohydrate in the list of vitamins and minerals in Annex II to Directive 2002/46/EC, allowing its use as a source of vitamin D in dietary supplements, since orally administered calcidiol has greater bioavailability compared with other compound forms [116].

### 4.5. Calcium

Calcium is available in a variety of dietary supplements, with the most common forms being calcium carbonate, calcium citrate, and calcium gluconate [117]. Calcium carbonate is inexpensive, widely available, and contains a relatively high proportion of elemental calcium (40%). However, it is less soluble in water and is therefore best taken with meals, as adequate gastric acid is required for its absorption. In contrast, calcium citrate is the most readily absorbed calcium supplement and can be taken with or without food, as its absorption does not depend on gastric acid levels. Compared with calcium carbonate, calcium citrate is more expensive and contains a lower proportion of elemental calcium (21%) [118,119]. It is also important to note that the simultaneous intake of iron and calcium impairs iron absorption. For this reason, the World Health Organization (WHO) recommends that the two be administered several hours apart, and that the total daily calcium intake be divided into three separate doses [120].

### 4.6. Iodine

Iodine naturally occurs in foods such as milk and seafood, primarily in the form of iodide salts, but also as iodate, molecular iodine, or monoatomic iodine incorporated into proteins. In addition, iodine is widely used to prevent deficiency, as an ingredient in supplements (e.g., sodium iodide, sodium iodate, potassium iodide, and potassium iodate), although salt iodisation remains the preferred strategy [121,122]. In any case, after ingestion in the form of iodide, iodate, or organically bound iodine, approximately 90% of the iodine is absorbed in the duodenum. The absorbed iodine is then transported to the thyroid gland via the sodium/iodide symporter (NIS) [123].

## 5. Multi-Component Food Supplements: Role of Micronutrient Interactions

Although the Directive 2002/46/EC of the European Parliament and of the Council on food supplements specifies which vitamins and minerals can be used in the manufacture of food supplements to ensure that they are safe and do not have any adverse physiological effects on the human body, their use is not entirely risk-free [121]. These products are not classified as medicines, for which manufacturers must demonstrate quality, efficacy (indication, dosage), and safety before they can be marketed. For dietary supplement products, there are no regulatory requirements to provide evidence for the criteria applied to medicinal products. Furthermore, it is also concerning that pregnant women may not always use these single- or multi-component supplements with the necessary caution or awareness; moreover, many women ignore nutritional and host factors [124]. Supplementation with multiple micronutrients can have a significant impact on pregnancy outcomes, as the micronutrient status of pregnant women depends on the adequacy of supplementation. There is no standard composition for multiple-component dietary supplements used during pregnancy, but most contain calcium, iodine, omega-3 fatty acids, zinc, and vitamins A and D, as well as iron and approximately twice the amount of folic acid found in multivitamins [125].

A study conducted in Poland concluded that most prenatal supplements fail to meet the recommended intake levels for individual nutrients [126]. Similar conclusions were reached in an analysis of the vitamin content of more than 180 commercially available dietary supplements, which found that prenatal preparations vary widely in composition, often contain only some of the essential vitamins, and frequently provide lower levels than recommended [127]. Furthermore, a recent study in the United States showed that of 20,547 dietary supplements—including 421 prenatal products—only 1 product (not prenatal) contained the recommended amounts of the six nutrients considered most important during pregnancy [128].

Beyond these findings, another concern regarding the micronutrient content of supplements is the potential for interactions between compounds within these products, as one substance can influence the absorption, metabolism, or elimination of another, or may modify its effects by acting synergistically or antagonistically at the same target site (e.g., an enzyme).

Micronutrient interactions can be synergistic, as seen with vitamins C and B2, which can enhance iron absorption, thereby supporting adequate maternal haemoglobin levels and reducing the risk of iron deficiency anaemia [129,130,131]. The synergy between folic acid and vitamin B12 is also critically important during pregnancy, as both are involved in homocysteine metabolism and DNA methylation. Consequently, there is an inverse relationship between elevated homocysteine levels and inadequate intake of folic acid and vitamin B12. Increasing evidence also suggests that disturbances in homocysteine metabolism, particularly due to excessive methionine, may contribute to the pathogenesis of neural tube defects (NTDs) [132,133].

On the other hand, some micronutrients can have antagonistic effects, such as combined iron and zinc supplementation, since iron can dose-dependently reduce zinc uptake. However, this effect depends on the source of intake, i.e., the form of the solution or food (dietary supplement, fortified food) [134,135,136,137]. Some studies also suggest that folic acid (especially at high doses) may reduce zinc absorption and impair its utilisation [138], but more recent evidence indicates that it does not adversely affect serum zinc concentrations during pregnancy [139,140]. Additional research has shown that calcium can inhibit iron absorption; however, the duration of supplementation is crucial, as long-term combined supplementation has not been shown to alter haematological values or iron status indicators [141,142]. Excessive magnesium intake may also negatively affect intestinal calcium absorption [143].

Moreover, compounds in these products can interact not only with each other but also with pharmaceutical agents or plant-derived chemicals (phytochemicals) [144]. It is undeniable that many women planning pregnancy or who are pregnant use prescription medications. Research has indicated that approximately 70% of pregnant women take at least one prescription drug [145].

Therefore, understanding the complex interactions between different micronutrients and other substances, and consequently ensuring the avoidance of potential adverse effects, is essential for developing optimal supplementation strategies.

## 6. Conclusions

Although micronutrient deficiencies during preconception, pregnancy, and lactation remain an issue, strategies such as supplementation and food fortification have improved micronutrient status. However, additional national and international studies are required to clarify the multiple effects of micronutrients and the determinants of their bioavailability. Furthermore, a comprehensive understanding of the factors affecting bioavailability, such as age, physiological status, nutrient status, and overall health in pregnant women, is essential. In addition, it is important to clarify the mechanisms of action of compounds found in supplements, to understand the synergistic and antagonistic interactions between components, and to determine their optimal timing and dosage in practice. In this regard, it is also necessary to establish EU-wide harmonised maximum levels for vitamins and minerals used in dietary supplements and fortified foods, thereby protecting consumers from excessive intake. Finally, it is also advisable to establish an EU-wide or more global system for monitoring and recording adverse effects of dietary supplements, which would further enhance consumer health protection. Such measures could support more informed, evidence-based decision-making regarding supplementation during pregnancy.

## Figures and Tables

**Table 1:** PRIs, AIs, ARs [57] and tolerable upper intake levels (ULs) [58] for micronutrients in pregnant women as determined by the EFSA.

Vitamins and Minerals (Unit of Measurement)	Age Group (Years)	AR ^(a)^	PRI ^(b)^	AI ^(c)^	UL ^(d)^
Folate (µg/day)	≥18 years	ND ^(6)^	NA ^(5)^	600 μg DFE ^(1)^/day	1000 μg/day ^(2)^
Vitamin B12 (µg/day)		ND ^(6)^	NA ^(5)^	4.5 μg/day	ND ^(6)^
Vitamin D (µg/day)		ND ^(6)^	NA ^(5)^	15 μg/day ^(4)^	100 μg VDE ^(3)^/day
Calcium (mg/day)	18–24 years	860 mg/day	1000 mg/day	NA ^(5)^	2500 mg/day
	≥25 years	750 mg/day	950 mg/day	NA ^(5)^	
Iron (mg/day)	≥18 years	7 mg/day	16 mg/day	NA ^(5)^	ND ^(6)^ (Safe level of intake of 40 mg/day)
Iodine (µg/day)		ND ^(6)^	NA ^(5)^	200 μg/day	600 μg/day

**(a) Average requirement (AR)**—The average requirement (AR) refers to the intake of a nutrient that meets the daily needs of half of the people in a typical healthy population. **(b) Population reference intake (PRI)**—The PRI level is the intake of a nutrient that is likely to meet the needs of almost all healthy people in a population. **(c) Adequate intake (AI)**—The AI level is used when there are not enough data to calculate an average requirement. An AI is the average nutrient level, based on observations or experiments, that is assumed to be adequate for the population’s needs. **(d) Tolerable upper intake level (UL)**—The UL is the maximum level of total chronic daily intake of a nutrient (from all sources) that is not expected to pose a risk of adverse health effects to humans. **(1) DFE: Dietary folate equivalent**—Naturally occurring food folates exhibit lower bioavailability compared with synthetic folic acid. To account for these differences, dietary folate equivalents (DFEs) have been established. For combined intakes of food folate and folic acid, DFEs are calculated as follows: μg DFE = μg food folate + (1.7 × μg folic acid). **(2) UL (folate)**—The ULs apply to folic acid, 5-MTHF-glucosamine, and L-5-MTHF-Ca. **(3) VDE: Vitamin D equivalent**—Different forms of vitamin D have different bioavailabilities. VDEs have been introduced to account for these differences. 1 μg VDE = 1 μg cholecalciferol (vitamin D3) = 1 μg ergocalciferol (vitamin D2) = 0.4 μg calcidiol monohydrate = 40 IU. This applies to calcidiol monohydrate at doses up to 10 μg/day. **(4) AI (Vitamin D)**—The Adequate Intake (AI) level for vitamin D refers to both ergocalciferol (vitamin D_2_) and cholecalciferol (vitamin D_3_). The dietary requirement is reduced and may, in some cases, be negligible when endogenous synthesis of vitamin D occurs. **(5) NA: AI/PRI level is not available**. **(6) ND: UL/AR cannot be determined.**

## Data Availability

No new data were created or analysed in this study. Data sharing is not applicable to this article.

## Abbreviations

The following abbreviations are used in this manuscript:

GDM	Gestational diabetes mellitus
PE	Preeclampsia
IUGR	Intrauterine growth retardation
LBW	Low birth weight
UL	Tolerable upper intake level
CaBP	Ca-binding protein
EFSA	European Food Safety Authority
WHO	World Health Organization
CRP	C-reactive protein
TSAT	Transferrin saturation
MMA	Methylmalonic acid
DRV	Dietary Reference Value
AI	Adequate intake
AR	Average requirement
PRI	Population reference intake level
NTD	Neural tube defect
DFE	Dietary folate equivalent
DHFR	Dihydrofolate reductase
DHF	Dihydrofolate
L-5-methyl-THF	L-5-methyltetrahydrofolate
UMFA	Unmetabolised folic acid
THF	Tetrahydrofolate
MTHFR	Methylenetetrahydrofolate reductase
SNP	Single-nucleotide polymorphism
MDA	Malondialdehyde
DBP	Vitamin D-binding protein
